# Pilot Study of Laser Doppler Measurement of Flow Variability in the Microcirculation of the Palatal Mucosa

**DOI:** 10.1155/2016/5749150

**Published:** 2016-06-01

**Authors:** Pierre Le Bars, Gaston Niagha, Ayepa Alain Kouadio, Julien Demoersman, Elisabeth Roy, Valérie Armengol, Assem Soueidan

**Affiliations:** ^1^Department of Prosthodonthics, UIC Odontologie, CHU de Nantes, 1 Place Alexis Ricordeau, 44042 Nantes, France; ^2^Department of Periodontology, Dental School of Brest, 22 Avenue Camille Desmoulins, 29238 Brest Cedex 3, France; ^3^Department of Pedodontics, UIC Odontologie, CHU Nantes, 1 Place Alexis Ricordeau, 44042 Nantes, France; ^4^Department of Restaurative Dentistry, UIC Odontologie, CHU Nantes, 1 Place Alexis Ricordeau, 44042 Nantes, France; ^5^Department of Periodontology, UIC Odontologie, LIOAD U-791, 1 Place Alexis Ricordeau, 44042 Nantes, France

## Abstract

*Background.* Histopathological alterations can arise when the denture-supporting mucosa experiences microbial and mechanical stress through the denture base and diagnosis of these diseases usually follows microvascular changes. Microcirculation measurement could allow for detection of such dysfunction and aid in the early diagnosis of palatal mucosa pathologies.* Materials and Methods*. We tested the sensitivity of laser Doppler for measuring the microcirculation of the palatal mucosa, assessing the median raphe (MR), Schroeder area (SA), and retroincisive papilla (RP). A Doppler PeriFlux 5000 System, containing a laser diode, was used. 54 healthy participants were recruited. We compare the measurements of PU (perfusion unit) using ANOVA test.* Results.* The numerical values for palatal mucosa blood flow differed significantly among the anatomical areas (*p* = 0.0167). The mean value of Schroeder area was 92.6 (SD: 38.4) and was significantly higher than the retroincisive papilla (51.9) (SD: 20.2) (*p* < 0.05), which in turn was higher than that of median raphe (31.9) (SD: 24.2) (*p* < 0.0001).* Conclusion.* Schroeder area appeared to have the greatest sensitivity, and vascular flow variability among individuals was also greatest in this region. We suggest that analysis of blood stream modification with laser Doppler of the palatal mucosa can help to detect onset signs of pathological alterations.

## 1. Introduction

The microcirculation of the palatal mucosa around the three anatomical areas tested here has not been previously investigated. The rationale for this study was based on the potential for blood stream measures around these areas to reveal early vascular alterations at the level of the connective tissue. Indeed, the clinical aspects of the mucosa do not always reflect the underlying histological features and can mask infraclinical modifications or alterations. Thus, in the absence of clinical parameters (erythema, pain, or oedema), the investigation of vascular microcirculation is of interest for detecting the onset stages of the pathology.

Denture-supporting mucosa is subjected to microbial and mechanical stress transmitted through the denture base, which can lead to development of certain diseases (e.g., prosthetic stomatitis). Histological and microvascular changes precede the clinical diagnosis of these conditions, and detection of such changes could potentially help in preventing the onset of these pathologies. Measurement of the microcirculation is useful for the early detection of palatal microvessel dysfunction and can help in the diagnosis of numerous palatal mucosa pathologies [1].

The laser Doppler flowmeter (LDF) is a noninvasive measure of capillary blood perfusion (blood flow, volume, and velocity). The laser Doppler measures the flow of blood cells inside a tissue without causing the slightest deterioration of the tissue. Blood cells moving within the volume illuminated by the beam will cause the light frequency [2] and are useful for measuring the microcirculation in healthy tissue in humans and nonhuman animals [3]. This approach was first used in the 1980s [4, 5] and has since been applied for many tissues, including the skin [6], tongue, and oral mucosa of healthy individuals [7], and the tooth [8], periodontal tissues [9–11], and the masseter muscle [12].

The efficacy of LDF used here has been previously employed in the study of various pathologies including wound healing in cutaneous sclerosis [13] and skin ischemia in rats [14], as well as diseases such as allergic reaction of the human nasal mucosa [15] and psoriasis [16]. Here, we aimed to measure the microcirculation of the healthy palatal mucosa at three specific points, measuring anatomical and histological variation, and to test the reproducibility and sensitivity of the LDF.

## 2. Materials and Methods

Fifty-four healthy students of the dental school at Nantes with no visible palatal mucosal abnormalities were recruited over a 7-month period. Participants were 20 to 26 years old and consisted of 32 men and 22 women, including 12 smokers (10 cigarettes/day). This study was performed in accordance with the ethical standards laid down in the 2002 Declaration of Helsinki and its later amendments.

One operator (GN) made the measurements using Laser Doppler Perfusion Monitor (PeriFlux System 5000; Perimed, Stockholm, Sweden) with a probe, latex particles, and a rotating disc.

The sensor emits monochromatic light at a 780 nm wavelength, which is absorbed by the mucosa. The range of light is 1 mm^3^ [17]. Students were seen in two sessions on a voluntary basis, and oral informed consent was obtained.

At the first session, a full clinical examination was conducted to determine the general and oral health status. Clinical parameters were also listed, including angle class, palate shape, and the clinical aspect of the palatal mucosa (colour, adhesion, surface, and appearance). Only participants considered healthy based on this examination were included. Exclusion criteria were hypertension, being on medication related to blood circulation, and a pronounced gag reflex.

**Figure 1:**
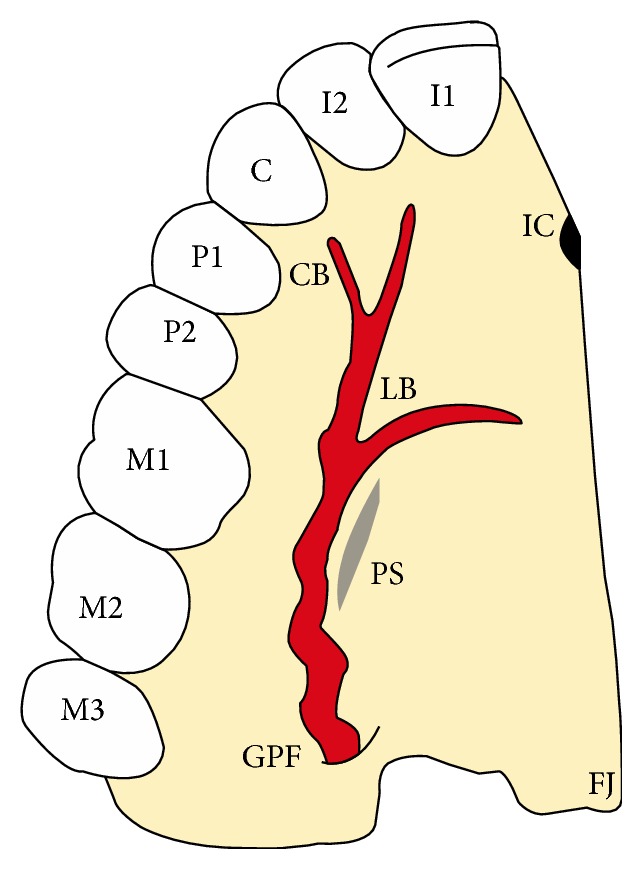
Diagram showing the branches of the artery great palate (GPA) and bony prominences palatine. The GPA emerges through the greater palatine foramen (GPF) from the maxillary artery, runs along the palatal spine (PS), and is divided into lateral branch (LB) and canine branch (CB), and leads to the incisive foramen (IC).

The first step consisted of the making of an alginate impression that was sent to the laboratory for gutter fabrication. To attach the probe during measurement, thermoformed gutter trays were made by the prosthetic laboratory of the dental school. These gutters, in transparent copolyester (acrylic resin; thickness 1 mm), were fitted with three brackets for attaching the probe during recording (Figure 1).

After completion of the gutters, three perforations were made for the probe supports. The three selected areas represent specific anatomical points: the retroincisive papilla, the median raphe, and the posterolateral Schroeder area. (Figure 1). We positioned our probe precisely halfway along the tangent between the distal surfaces of the first molar to the median raphe.

**Figure 2:**
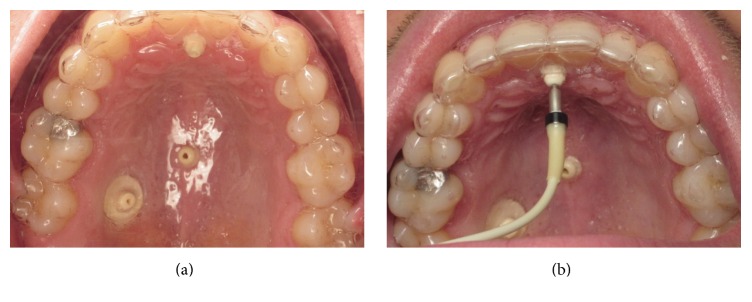
Thermoformed trays in the mouth (a) with three brackets for attaching the probe during recording (retroincisive papilla, median raphe, and Schroeder area) (b).

During the second session, The LDF was calibrated before each data collection session, with a colloidal suspension of latex microparticles (Perimed Mobility Standard), the flow of which corresponds to the value 250 (±15). All recordings were made in the same place at room temperature, but even the temperature of the oral cavity can be somewhat variable between individuals. They were placed on a dental chair in a comfortable position, half-inclined. After the patient rested for 5 min in a prone position, these measures allow us to include patients checking the normality of blood pressure. These measures denote the normality of the pulse and saturation and represent a criterion of selection. Finally, the measurements were performed with the probe that had been previously stabilized by the support. Each recording lasted for 3 min (Figure 2).

The statistical analysis involved paired-samples *t*-tests. One-way analyses of repeated measures of variance (ANOVA) were applied to compare three areas tested under xlstats®. *p* < 0.05 was taken as indicating statistical significance. According to the central theorem limit, the distribution of the mean of the sample greater than 30 patients would authorize or permit the use of Student's *t*-test to compare each mean of each group of data.

## 3. Results

**Figure 3 fig3:**
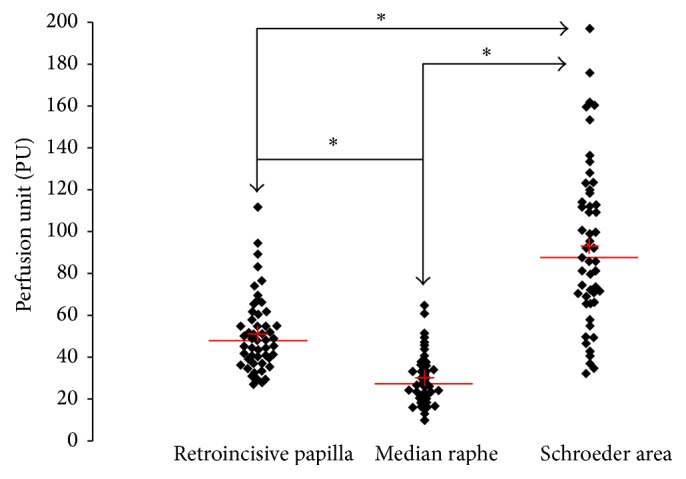
Scatterplot of individual measured values in the three zones. ^*∗*^Statistically significant difference between the different anatomical areas (*p* value < 0.05). We found that the average value at the Schroeder area (PU = ±92) was significantly higher than that measured at the retroincisive papilla (PU = ±51.92) (*p* < 0.05), which is higher than the median raphe (PU = ±31.97) (*p* < 0.0001). (The red line shows the average blood flow measurements in the three study areas.)

**Figure 4 fig4:**
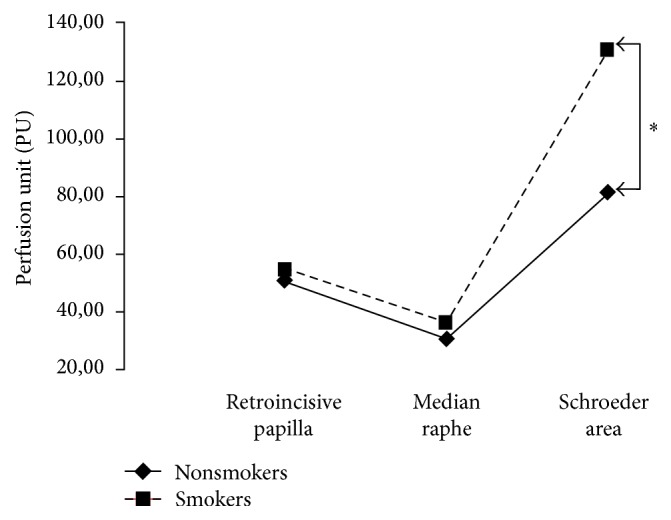
Mean comparisons of PU measure between smokers and nonsmokers patients in Schroeder area. The average values of the three zones were generally higher in smokers than in nonsmokers. However, this difference was only statistically significant for the Schroeder area (*p* = 0.005)^*∗*^.

**Figure 5:**
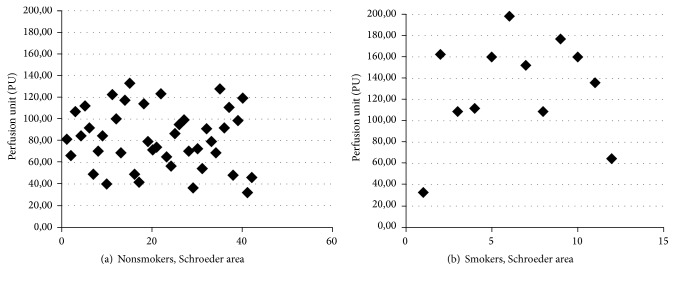
Scatterplot values in Schroeder area in nonsmokers (a) and smokers (b).

Differences in the blood flow to the palatal mucosa were expressed as a percentage of the PU value (Figures 2 [Fig fig3] [Fig fig4]–5).

In the first group (*n* = 54), ANOVA analysis of the repeated measures for the entire group (*n* = 54) (12 smokers + 42 no smokers) identified a statistically significant difference between the tested anatomical areas one to one (*p* = 0.0167). We found that the mean value of Schroeder area was (92.6) (SD: 38.4) and was significantly higher than the retroincisive papilla (51.9) (SD: 20.2) (*p* < 0.05), which in turn was higher than that of median raphe (31.9) (SD: 24.2) (*p* < 0.0001) (Figure 3).

In the second group of only no smokers (*n* = 42), the mean measured values for the three were 81.6 (SD: 27.5) (Schroeder area), 51.1 (SD: 18.4) (retroincisive papilla) (*p* < 0.0001), and 30.7 (SD: 26.4) (median raphe) (*p* < 0.0001).

In the third group of only smokers (*n* = 12), we also found that the average values of the three zones were generally higher in smokers than in nonsmokers. However, this difference was statistically significant only for the Schroeder area 130.9 (SD: 47.3) (*p* = 0.005) (Figure 4).

Closer analysis of the dispersion of values for the Schroeder area revealed a dense concentration of the measures in nonsmokers (Figure 5(a)), compared with more dispersed values in smokers (Figure 5(a)).

## 4. Discussion

A removable prosthesis exerts pressure on the oral mucous membranes and in particular on the palatal mucosa during mastication. Under these conditions, the blood supply of the mucous membrane is then modified at histological level before the apparition of clinical signs. The question for us was whether we have the ability to detect and measure these vascular histological changes before the apparition of clinical signs.

To answer this question, we wanted to test the reliability and laser Doppler sensitivity level of vascularization of the palatal mucosa. We conducted, on a sample of 54 healthy patients, measurements at three sites in the palatal mucosa (retroincisive papilla, the median raphe, and Schroeder area).

To validate our measures, several parameters must be apprehended as the depth of the palatal mucosa and the influence of the age of patients.

For the depth, five studies have recently involved the palatal mucosa (in longitudinal and transverse planes) using various techniques. One study examined (transversely) 34 hemimaxillae of cadavers (13 men and 4 women; mean age: 57.2 years). The thicknesses of the palatal mucosa and the* lamina propria* including the epithelium were measured at three points, starting from the alveolar crest, at intervals of 4 mm and with the aid of Adobe Photoshop®. The thicknesses of the palatal mucosa increase from the alveolar crest toward the midpalatal suture. Conversely, the thicknesses of the* lamina propria* including the epithelium at these same positions decrease toward the midpalatal suture [18].

Kolliyavar et al. [19], Anuradha et al. [20], and Yaman et al. [21] measured (longitudinally) the gingival margin and palatal line area. A bone-sounding method using a periodontal probe was used to assess the thickness of the palatal mucosa at 15 measurement sites, and the difference in mucosal thickness between the groups was determined. The mean thickness of the palatal masticatory mucosa ranged from 2.0 to 3.7 mm.

Cho et al. [22] used light microscopy to investigate the longitudinal depth from the surface of the palatal mucosa. The thickness of the epithelium and* lamina propria* of the palatal mucosa was measured (from the canine distal area to the first molar distal area) at three positions (starting from 3 mm below the alveolar crest and in 3 mm intervals) along the path of the palatine artery. The mean depth from the surface of the palatal mucosa to the greater palatine artery decreased from the canine distal to the first premolar distal but again increased towards the posterior molar. The mean length from the alveolar crest to the greater palatine artery, however, increased toward the posterior molar.

Our measurements are effective to a depth of 1 mm, which corresponds to a* lamina propria* volume of only 1 mm^3^. Indeed, under this condition, the thickness of the palatal mucosa was not affected in our results.

Secondly, the effects of aging were not consistently reported in these three studies. Although Kolliyavar et al. [19] and Anuradha et al. [20] found that younger participants had thinner mucosa than older participants, Yaman et al. [21] detected no significant difference between age groups. Anuradha et al. [20] also found that, within the same age group, females had thinner mucosa than males whereas Yaman et al. [21] identified no differences according to gender or body mass index. These conflicting findings are likely to result from study differences in age, ethnicity, body mass index, varying measurement methods, and the placement of measurement points.

If we compare these study groups to those of our study, our participants were relatively healthier and younger (mean 23 years) and had a lower weight mean (65 kg).

Another interesting aspect is to compare the measures obtained by laser Doppler between the skin and palatal mucosa; the buccal epithelium is relatively finer than the skin epidermis [23]. Despite this difference, the signal obtained from contact with the oral mucous membrane is less pronounced than that of the skin of the cheek. The density of capillaries varies according to the anatomical zones of the human body, so at the level of the bowel the average density of the capillaries is about 50 capillaries by square millimetre of mucous surface [17]. Another explanation is the fact that the vascular network of oral tissues is less rich than that of the skin [24]. Although it is possible that the presence or absence of epithelial fingering can change the morphology of the epithelium, we propose that our measurements were not influenced by these histological characteristics because the operating range of laser Doppler signal exceeds the thickness of the epithelium.

Concerning the precision of the measures, in our study, for better recording probe support, an in-mouth stabilizing patch was developed. The thickness of the gutters (<2 mm) does not influence measures of blood flow in the mucosa [25]; indeed, amelioration of recording reliability over time could be verified by using this approach.

Another parameter is that the fluctuation in the lower frequencies at 0.1 Hz depends on the sympathetic nervous system at the level of the blood flow to the skin [26]. Under the same conditions for the palatal mucosa, the precision of our measures can be limited. This parameter is much more transient at the level of the oral mucous membrane than at the level of the skin. Histamine is quickly eliminated at the level of the vascular buccal network [24]. Furthermore, at the level of the skin, the proportion of the nerve network that is influenced by histamine is large and with a prolonged effect [5, 8, 9]. It seems that, in the case of our experiment, this parameter did not influence the result because every measurement was conducted on healthy participants after 30 min of complete rest.

Our anatomical and histological findings enabled us to demonstrate a significant difference in the microcirculation in the three areas. The flow was much more important in the Schroeder area compared to the retroincisive papilla and was significantly higher than the median raphe. The average flux values were higher in the Schroeder area, differences that could have been revealed only by the sensitivity of the laser Doppler device.

### 4.1. Smoking and Nonsmoking Patients

It is well established that smoking modifies the vascular network, with a hyperaemic response in the palatal mucosa of smokers compared to nonsmokers. In the present investigation, the increased PU value of smokers was probably the result of a local vascular vasoconstriction effect, particularly at the most peripheral portion of the mucosa. The elevation of microcirculation in the palatal mucosa serves as a trigger for angiogenesis of the palatal vascular plexus, and our results confirm this other research [27].

In our study, when comparing smokers to nonsmokers, we observed increased microcirculation in smokers, particularly in the connective tissue of the Schroeder area. A local effect of smoking on the palatal mucosa is well established [28, 29] and can potentiate interaction of the flow, particularly in the Schroeder area. There we propose that the Schroeder area would likely be the most informative/interesting zone in which we measure the flow and compare findings between participant groups. Concerning our sample, we took into account interindividual variability including general condition, similar hemodynamic and ambient temperatures [30], age, gender, site, posture, and ethnicity. Other parameters that can influence intraindividual comparisons include menstrual cycle, circadian rhythm, and physical activity whereas mental activity does not influence the results [31].

The relatively homogeneous age of the participants in this study (22 to 26 years old) means that age had little influence on our results [32]. The flexibility of blood vessels is reduced with increased age. In the same way, a decrease in the thickness of the oral mucous membrane occurs with aging. Another aspect is fibrosis of connective tissue in the* lamina propria*, which appears with aging and can influence the measures [33].

### 4.2. Influence of a Covered Removable Prosthetic Denture on the Palatal Mucosa

In an interesting study [34], laser Doppler was used to investigate recovery after removal of a partial denture (bilateral posterior maxilla edentulous) in the palatal mucosa of the Schroeder area [34]. The results of this referenced study did not reveal significant changes in the blood flow of the palatal mucosa in the Schroeder area over a 12-minute period. It seems that wearing a resin partial denture modifies the vascularization of the palatal mucosa in the area of Schroeder, independently of disease. It would also seem that bearing a partial removable prosthesis in resin increases the stream of the vascularization of the palatal mucous membrane of the zone of Schroeder, independently of any clinically detectable sign of local pathologies.

It has been suggested that a denture on tissue surfaces must irritate the mucosa, and the Schroeder area is often implicated in the prevalence of denture-related stomatitis [35]. Changes to blood flow at the level of the palatine mucous membrane are not initially clinically detectable, and for this reason laser Doppler can constitute an appropriate tool for diagnosis or early investigation of certain pathologies (e.g., burning mouth syndrome [9]) that present considerable difficulties with visual detection. Recently, after clinical observations, a new laser Doppler perfusion tool, PeriScan PIM II, has been used to evaluate the distribution maps of blood flow corresponding to specific areas [36].

## 5. Conclusion

Application of LDF to measure blood flow in the palatal mucosa can differentiate three anatomical areas, with measurement of the Schroeder area being the most sensitive. The incidence of the mucosa thickness variability highlights the value of routine measurement of microcirculation by laser Doppler in patients presenting mucosal lesions. This study represents a technical advance and opens new possibilities for investigating diseases of the palatal mucosa, including tissue changes related to dentures, such as denture-related stomatitis. The influence of certain technical impressions or treatments (e.g., radiotherapy or bisphosphonates) can alter the subepithelial microcirculation, and these alterations can be measured at the blood vessel level using LDF. This possibility also applies for more systemic diseases such as diabetes. Therefore, further investigation will be required to better establish the clinical potential of LDF. We currently are extending this study of the palatal mucosa blood microcirculation in the same areas of denture wearers with clinically healthy mucosa and those with different denture-related stomatitis diseases.
